# A software package for simple and rigorous survival machine learning analysis in biomedical research

**DOI:** 10.64898/2026.08.05.26359034

**Published:** 2026-08-10

**Authors:** Alyssa F. Pybus, Junhao Qiu, Paulo Cilas Morais Lyra, Khai Dang, Isis Narvaez-Bandera, Tosin Jolaogun, Jeremy Goecks

## Abstract

Survival analysis is a fundamental technique in biomedical research for modeling time-to-event data. It enables the identification of prognostic factors in disease, compares survival outcomes across treatment groups, and performs targeted treatment selection. A variety of machine learning (ML) approaches to survival analysis have emerged to complement classical statistical methods, especially for high-dimensional datasets with complex, nonlinear interactions between features. However, using survival ML methods requires addressing challenges such as censoring-unaware evaluation, overfitting, selecting performance metrics, and data leakage. To address these and other difficulties in using survival ML models, we developed the mlsurv software package. mlsurv is an open-source Python package built around three major design principles: 1) methodological rigor, including evidence-based model selection, leakage-free pipelines, and multi-metric evaluation, 2) multi-scale evaluation and interpretation, including population and subpopulation evaluation, patient-level explanations, and feature analysis, and 3) automated trust and transparency, including limitation flagging and TRIPOD+AI-aligned reporting. mlsurv bundles ten models spanning linear, ensemble, kernel, and deep learning families within a unified software package. To our knowledge, mlsurv is the first package to span the complete survival ML workflow from automated model recommendation through TRIPOD+AI reporting and individual patient explanation. We demonstrate mlsurv on the Chowell immunotherapy cohort (n=1,479). We found that overall survival (OS) was more predictable than progression-free survival (PFS) (concordance of 0.73 vs 0.67). Albumin was a top feature for both endpoints but dominated OS prediction, whereas tumor mutational burden rose to co-lead PFS prediction. Survival models matched the response-trained LORIS clinical score on PFS prediction and exceeded it on OS. mlsurv enables biomedical researchers to conduct rigorous, multi-model survival analysis and benchmarking using minimal code with default best practices rather than implementing custom scripts and methodological safeguards from scratch.

## Keywords

Survival analysis, machine learning, predictive modeling, predictive machine learning, time-to-event analysis

## Background

Survival analysis underpins prognosis, treatment selection, and trial design across biomedical research and clinical practice. Survival methods analyze the time until an event of interest (e.g., progression, death) and account for censored observations where event time is unknown beyond a subject's last follow-up. Survival and hazard functions summarize event risk over time for each patient and are traditionally modeled with covariates via Cox proportional hazards regression (1). However, modern biomedical datasets often feature high dimensionality, complex feature interactions, and non-proportional hazards that Cox regression does not accommodate.

Researchers are increasingly adopting machine learning (ML) for survival prediction in modern complex biomedical datasets (2–7). However, executing robust ML survival analysis requires overcoming several challenges. First, ML models must be selected, tuned, and evaluated to ensure methodological rigor and avoid leakage as well as overfitting that can inflate reported performance (8). Second, comprehensive model evaluation and interpretation are required at different levels of observation, from subpopulations to individuals, and across features and their interactions. Third, best practices are needed to promote trustworthiness of the analysis, including verifying model assumptions and dataset adequacy, enabling reproducibility, and comprehensive reporting. The growing use of artificial intelligence (AI) to perform biomedical analyses further amplifies these concerns, as such tools may omit steps or introduce errors (9,10).

Existing software tools address isolated parts of this workflow, but none provide an integrated pipeline spanning model selection through patient-level explanation. Current software tools perform parametric modeling (11,12), machine learning models (12–14), deep learning (15,16), and hyperparameter tuning (14,17), but rarely encompass a full survival analysis from start to finish. This fragmentation carries real methodological risks. Data leakage remains the most common error in ML research, affecting a substantial fraction of published studies (18,19). Model selection itself lacks clear guidance, as no single model class reliably dominates across survival datasets (20,21). For example, a recent meta-analysis of 21 cancer survival studies found extensive between-study heterogeneity in ML vs. CoxPH performance (22), suggesting task-specific model benchmarking is essential for performance optimization. Despite this evidence, ad hoc analyses routinely commit to a specific model without systematic comparison.

To bridge these gaps and enable rigorous, comprehensive ML-based survival analysis, we have developed `mlsurv`, an open-source software package for robust, end-to-end ML survival modeling. `mlsurv` is implemented in Python using three design principles: methodological rigor, multi-scale evaluation and interpretation, and automated trust and transparency (**Table 1**). `mlsurv` bundles ten survival analysis models from multivariate Cox proportional hazards (CoxPH) to deep learning models behind a unified tool with built-in methodological safeguards. Each experiment is organized as a single end-to-end pipeline with discrete analytical stages: leakage-protected preprocessing, evidence-based model recommendation, hyperparameter tuning, prospective patient risk scoring with bootstrap confidence intervals, population-, subpopulation-, and patient-level interpretation via SHAP explanations and feature-interaction analysis, and TRIPOD+AI (Transparent Reporting of a multivariable prediction model for Individual Prognosis Or Diagnosis, plus Artificial Intelligence) reporting. We validate `mlsurv` by reproducing published survival analyses across multiple models and datasets. We then demonstrate the full `mlsurv` workflow on the Chowell immunotherapy cohort (23) as an in-depth case study to demonstrate the complete workflow. In this cohort, survival-trained models match or exceed the performance of the response-trained LORIS biomarker, highlighting the differences between overall and progression-free survival prediction as well as prognostic versus predictive biomarkers.

**Table 1. Three design principles for biomedical survival machine learning and their implementation in `mlsurv`.**

| Design Principle | Risk Addressed | `mlsurv` Solution |
|---|---|---|
| Methodological rigor | Overfitting, data leakage, and evaluation errors that inflate reported performance | Data-driven model selection guidance, leakage-free preprocessing, and censoring-aware multi-metric evaluation |
| Multi-scale evaluation and interpretation | Summary metrics obscure subpopulation disparities, feature drivers, and individual risk factors | Evaluation across population, subpopulation, and individual scales; feature importance, interaction, and local explanation analysis |
| Automated trust and transparency | Undetected statistical issues, no audit trail, and gaps in TRIPOD+AI compliance | Automated limitation flagging, structured interactive reporting, and TRIPOD+AI compliance scaffolding |

## Implementation

The sections below highlight the main features and innovations of `mlsurv`. Detailed software reference, usage examples, and implementation notes for `mlsurv` are available at https://github.com/goeckslab/mlsurv/tree/main/docs.

### Overview

`mlsurv` makes it simple to perform survival ML analyses by requiring minimal code to perform a complete analysis using best practices. The package provides a set of high-level commands for an entire survival machine learning workflow through pipeline configuration, training, evaluation, prediction, analysis, and reporting (**Fig 1**). An entire workflow can be completed with just three commands – `setup()`, `run()`, and `generate_report()` – while optional methods are available for customizable workflows and in-depth analysis. `setup()` configures the preprocessing pipeline, cross-validation strategy, and train/test split. `run()` handles the full modeling cycle from training and tuning through evaluation and feature analysis, returning structured results. Lastly, `generate_report()` assembles all results into an interactive report. **S1 Table** lists the full set of high-level functions provided by `mlsurv`.

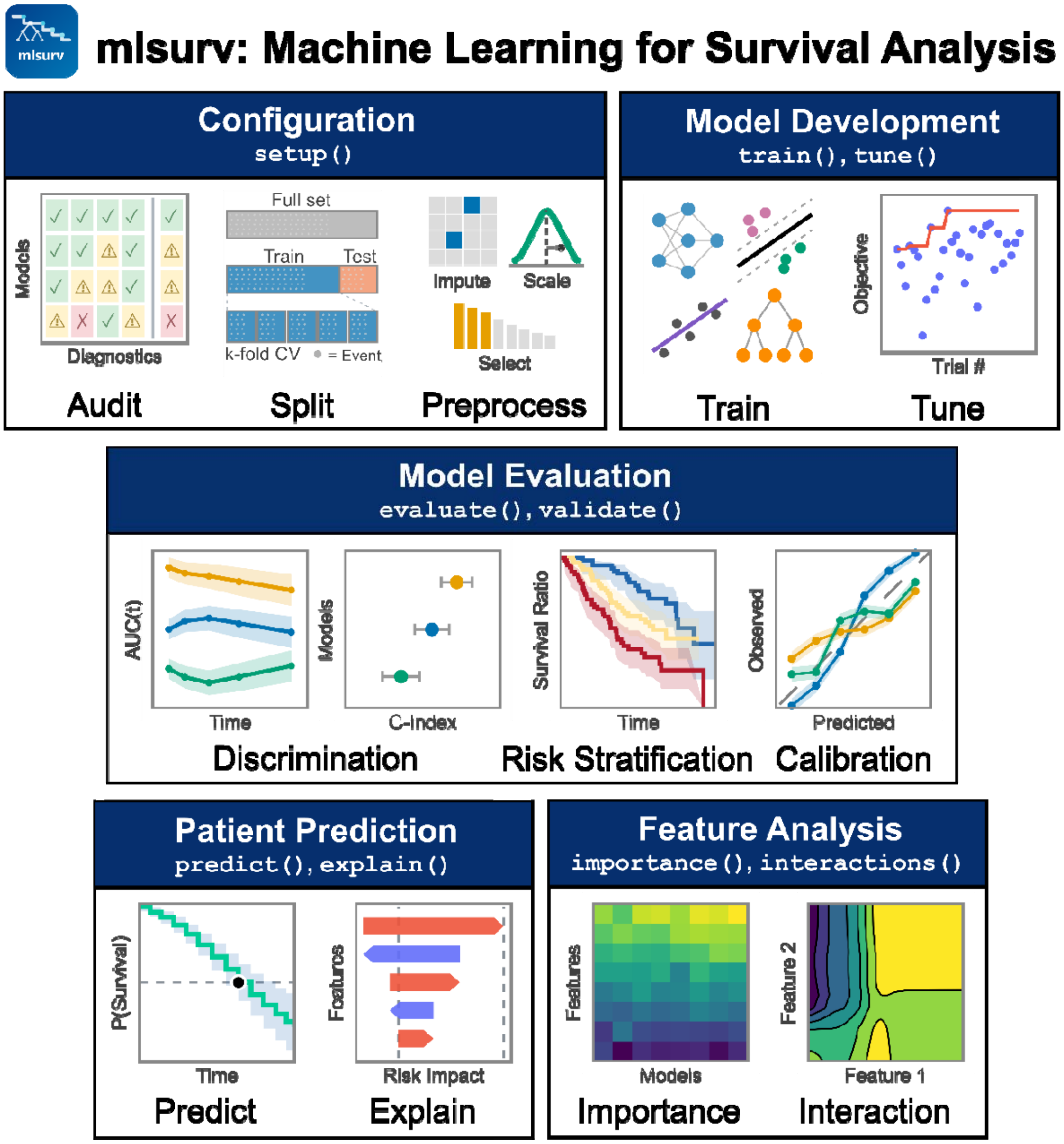

**Fig 1. Overview of the `mlsurv` software package.** Stages of a standard survival ML workflow are listed in each table alongside their corresponding `mlsurv` methods. During configuration, the `setup()` command audits the dataset to recommend appropriate models, splits the dataset into test/train and CV folds, and sets preprocessing steps. In model development, the `train()` command trains a variety of survival ML models and `tune()` conducts hyperparameter optimization. Model evaluation is performed by the `evaluate()` and `validate()` commands to calculate discrimination and calibration metrics as well as conduct risk stratification. Survival prediction on new patient data is performed with the `predict()` command. SHAP risk explanation by feature is computed by `explain()`. Feature analysis is performed with the `importance()` and `interactions()` commands.

`mlsurv` integrates ten survival models spanning four families of analysis: linear, ensemble, kernel, and deep learning (**Table 2**). All models share a unified interface for fitting, prediction, survival function estimation, scoring, and feature importance extraction.

**Table 2. Survival models available in `mlsurv`.**

| Model | Abbrev. | Family | PH Assum. | Source | Reference |
|---|---|---|---|---|---|
| Cox Proportional Hazards | CoxPH | Linear | Yes | scikit-survival (12) | Cox (1972) (1) |
| Elastic Net Cox Regression | CoxNet | Linear (regularized) | Yes | scikit-survival (12) | Simon et al. (2011) (24) |
| Weibull Accelerated Failure Time | Weibull AFT | Linear (parametric) | Yes | lifelines (11) | Kalbfleisch & Prentice (2002) (25) |
| Random Survival Forest | RSF | Ensemble | No | scikit-survival (12) | Ishwaran et al. (2008) (26) |
| Gradient Boosted Survival Analysis | GBSA | Ensemble | Yes* | scikit-survival (12) | Hothorn et al. (2006) (27) |
| XGBoost Cox Regression | XGBoost-Cox | Ensemble | Yes | XGBoost (13) | Chen & Guestrin (2016) (13) |
| XGBoost Accelerated Failure Time | XGBoost-AFT | Ensemble | No | XGBoost (13) | Barnwal et al. (2022) (28) |
| Fast Survival Support Vector Machine | FSSVM | Kernel | No | scikit-survival (12) | Pölsterl et al. (2015) (29) |
| DeepSurv | DeepSurv | Deep Learning | Yes | pycox (15) | Katzman et al. (2018) (30) |
| DeepHit | DeepHit | Deep Learning | No | pycox (15) | Lee et al. (2018) (31) |

*GBSA assumes proportional hazards (PH) only with the default Cox partial-likelihood loss.

## Data and Pipeline Configuration

`mlsurv` loads survival data from tabular files and validates event encodings, rejecting non-positive times. `setup()` configures the train/test split, cross-validation (CV) strategy, and preprocessing components. Default settings use a 30% event-stratified test split, five-fold CV, and Harrell's concordance index (C-index) (32) for model ranking. Advanced users can supply custom-built preprocessors, models, and evaluation metrics. Automated diagnostics are integrated to identify common constraints in survival modeling, including low events-per-variable (EPV), outcome-associated missingness, and Cox-family assumption violations for proportional hazards (33) or log-hazard linearity (34). `mlsurv` also generates tiered model recommendations based on published best practice EPV and sample size thresholds (34–37).

Pre-processing follows a fixed stage order: imputation, scaling, and feature selection. The full preprocessing pipeline is fitted independently within each fold so that CV scores reflect out-of-sample performance of the complete pipeline, preventing the inflated estimates that arise when preprocessing uses leaked information from validation folds.

## Model Development

`mlsurv` provides two complementary approaches for building survival models. `run()` executes the full model development pipeline in a single call: training, tuning, test set evaluation, and bootstrap confidence intervals. Alternatively, the individual commands—`train()`, `tune()`, `evaluate()`, and `bootstrap()`—support incremental workflows that can be used to compare default and tuned models or add models to an existing comparison. By default, all models share identical preprocessing, CV folds, and metrics for fair head-to-head comparison.

Hyperparameter tuning uses Optuna's Tree-structured Parzen Estimator (TPE) for Bayesian optimization (17) with default EPV-aware search space narrowing and convergence detection. The search space spans both model hyperparameters and preprocessing parameters, enabling joint optimization across the full model development pipeline. A single `random_state` parameter seeds all underlying frameworks (scikit-learn (14), XGBoost (13), PyTorch (38)) for end-to-end reproducibility.

## Model Evaluation

The `evaluate()` command measures the models' performance on the held-out test set across discrimination and calibration metrics designed for right-censored data (**Table 3**). The scoring metric used for model selection is configured during the `setup()` command. The `bootstrap()` command derives confidence intervals by repeatedly measuring model performance on the test set through resampling (39). Pairwise model comparisons via the Kang nonparametric z-test (40) assess whether performance differences are statistically significant, and quantile-based risk stratification evaluates clinical utility via log-rank (41) and restricted mean survival time (RMST) (42,43) comparisons.

**Table 3. Evaluation metrics for right-censored survival data produced by `mlsurv`.**

| Dimension | Metric | Description | Reference |
|---|---|---|---|
| Discrimination | Harrell's C-index | Concordance probability between predicted risk and observed outcome | Harrell et al. (1982) (32) |
| Discrimination | Uno's IPCW C-index | Censoring-adjusted concordance using inverse probability of censoring weighting | Uno et al. (2011) (44) |
| Discrimination | Antolini's C-index | Time-dependent concordance based on cumulative/dynamic AUC | Antolini et al. (2005) (45) |
| Discrimination | AUC(t) | Time-dependent area under the receiver operating characteristic curve | Uno et al. (2007) (46) |
| Calibration | Calibration slope & intercept | Agreement between predicted and observed survival probabilities | Steyerberg et al. (2010) (47) |
| Calibration | Greenwood-Nam-D'Agostino test (GND) | Goodness-of-fit test comparing predicted survival to Kaplan-Meier estimates | Demler et al. (2015) (48) |
| Calibration | Brier score (t) | Mean squared error between predicted survival probabilities and observed outcomes at a given time | Graf et al. (1999) (49) |
| Calibration | Integrated Brier Score (IBS) | Brier score integrated over the follow-up period | Graf et al. (1999) (49) |

When cohort subpopulations are declared at `setup()`, `mlsurv` evaluates each model within every subpopulation across all metrics. Event by subpopulation stratification in splits and CV folds preserves subpopulation proportions to prevent imbalanced evaluation, and subpopulations with fewer than 30 events are flagged. When models show poor calibration, users are advised to test different available calibration strategies during model training.

For prospective use, the `predict()` command generates risk scores, survival probabilities at user-specified time points, and median survival times with bootstrap confidence intervals for new patient data beyond the existing dataset.

## Feature Analysis

Feature analysis quantifies which variables drive predictions and how they interact, spanning global importance across the cohort to local explanations for individual patients.

Three complementary methods quantify feature importance: permutation importance with significance testing via the permuted response method (50,51), applicable to all models; SHAP (Shapley Additive exPlanations) values, which decompose each prediction into per-feature contributions and aggregate to both global rankings and individual explanations (52); and Cox coefficients and hazard ratios with confidence intervals, for models that expose them.

Interaction analysis identifies pairs of variables whose joint influence on predicted risk differs from the sum of their separate contributions. The H-statistic quantifies interaction strength via partial dependence for all nonlinear models (53), and SHAP interaction values decompose pairwise contributions per-sample for tree-based models (52,54). Two-dimensional partial-dependence surfaces visualize the joint effect of top pairs.

At the individual level, `explain()` decomposes a prediction into per-feature SHAP contributions, rendered as an interactive waterfall plot. Explanations apply to new patient data as well as the evaluation set.

### Validation and Benchmarking

External validation on independent cohorts is essential for assessing whether a model generalizes beyond its training population (19,55). The `validate()` command evaluates all trained models on one or more held-out datasets, computing the same discrimination and calibration metrics used during model training.

Beyond external validation, `mlsurv` provides comparative benchmarking to quantify the added value of a new model over one or more established baselines such as clinical scores or single biomarkers. This is done by using the `benchmark()` method to compute delta C-index, Net Reclassification Improvement (NRI), and Integrated Discrimination Improvement (IDI) with bootstrap confidence intervals and significance testing (56,57).

### Comprehensive and Interactive Reporting

Throughout the analysis pipeline, `mlsurv` automatically evaluates fourteen limitation flags that surface potential methodological concerns (**S2 Table**). Each flag explains the limitation, why it matters clinically or statistically, and a concrete suggested action. The flags span sample adequacy, data quality, model diagnostics, and study design. A three-tier warning system controls visibility: methodological warnings always surface to the user, suggestion warnings appear once per issue, and internal warnings are suppressed by default but available for debugging.

The `generate_report()` command produces a comprehensive interactive HTML report assembling all analyses, with visualizations rendered as interactive Plotly figures and also available as standalone methods for custom analyses (**S3 Table**). For individual patient reporting, the `patient_report()` command produces an HTML with a predicted survival curve and summary, a SHAP feature explainer waterfall plot, and training set nearest-neighbor tables. Lastly, `export_results()` writes all results to spreadsheet, CSV, or Parquet formats, and `save_learner()` serializes the full learner state for later reuse.

The report includes a pre-populated TRIPOD+AI compliance checklist mapped against the 52-item reporting standard for AI-based prediction models (19). Of the 52 items, 18 are auto-filled from analysis metadata, 10 are scaffolded with guidance referencing the relevant report section, 22 are flagged as researcher-provided, and 2 are marked as not applicable. This simplifies the user's reporting burden while ensuring that the computable items are populated accurately and consistently.

## Results and Discussion

### Three Design Principles for Biomedical Survival Machine Learning

We designed `mlsurv` around three core design principles that address key requirements in survival machine learning analysis. **Methodological rigor** ensures best practices for model development from pre-processing through hyperparameter tuning to protect against overfitting and data leakage. **Multi-scale evaluation and interpretation** enables comprehensive performance assessment of models across the full dataset, stratified subpopulations, and individual patients. Interpretation of the features used by a model or models is done using global importance and local explanations. **Automated trust and transparency** grounds `mlsurv`'s pipeline in established reporting standards through a tiered warning system for dataset and methodological limitations, structured outputs via an interactive report and comprehensive spreadsheet of results, and TRIPOD+AI reporting compliance. Seven tutorial vignettes on public datasets demonstrate one or more of these principles in use (**Table 4**). Vignette 7 demonstrates all three principles in an end-to-end analysis of the Chowell immunotherapy cohort (23) and its LORIS response scores (58).

**Table 4. Vignettes showcasing `mlsurv` capabilities.**

| # | Vignette Title | Dataset(s) | Runtime |
|---|---|---|---|
| 1 | Quickstart | METABRIC (n=1,903) (59) | ~1 min |
| 2 | Workflow Tour | GBSG2 (n=686) (60), Rotterdam (n=2,982) (61) | ~5 min |
| 3 | Benchmarks | SUPPORT (n=8,873) (62), METABRIC, FLCHAIN (n=6,521) (63), GBSG2, Veterans (n=137) (25) | ~10 min |
| 4 | Custom Components | TCGA BRCA (n=1,071) (64) | ~3 min |
| 5 | Advanced CV & Tuning | Rotterdam, GBSG2 | ~3 min |
| 6 | Tool Comparison | GBSG2, METABRIC | ~5 min |
| 7 | Immunotherapy Case Study | Chowell (n=1,479) (58,65) + 7 external cohorts | ~15 min |

#### Principle 1: Methodological Rigor

Survival ML models frequently overfit on their training cohort and fail to generalize to unseen cohorts. Overfitting can result from a mismatch between model complexity and dataset characteristics, information leakage across data splits, or inappropriate performance evaluation. `mlsurv` addresses these concerns with pre-training diagnostics, leakage-free pipelines, and censoring-aware discrimination and calibration metrics for model evaluation. With these safeguards, we used `mlsurv` to reproduce published benchmark C-indices with mean absolute deviation below 0.01 using default settings in Vignette 3 (**Table 5**).

**Table 5. `mlsurv` performance against published concordance indices on five public datasets.**

| Dataset | Model | mlsurv | Target | Gap | Source |
|---|---|---|---|---|---|
| GBSG2 | CoxNet | 0.668 | 0.669 | -0.001 | scikit-survival docs |
| GBSG2 | RSF | 0.690 | 0.691 | -0.001 | scikit-survival docs |
| Veterans | FSSVM | 0.724 | 0.720 | +0.004 | scikit-survival docs |
| METABRIC | CoxPH | 0.631 | 0.628* | +0.003 | Kvamme 2019 (66) |
| METABRIC | DeepSurv | 0.639 | 0.636* | +0.003 | Kvamme 2019 (66) |
| METABRIC | RSF | 0.662 | 0.649* | +0.013 | Kvamme 2019 (66) |
| METABRIC | DeepHit | 0.673 | 0.675* | -0.002 | Kvamme 2021 (15) |
| FLCHAIN | CoxPH | 0.794 | 0.790* | +0.004 | Kvamme 2019 (66) |
| FLCHAIN | DeepSurv | 0.796 | 0.790* | +0.006 | Kvamme 2019 (66) |
| FLCHAIN | RSF | 0.785 | 0.784* | +0.001 | Kvamme 2019 (66) |
| FLCHAIN | DeepHit | 0.794 | 0.791* | +0.003 | Kvamme 2021 (15) |
| SUPPORT | CoxPH | 0.595 | 0.598* | -0.003 | Kvamme 2019 (66) |
| SUPPORT | DeepSurv | 0.582 | 0.611* | -0.029 | Kvamme 2019 (66) |
| SUPPORT | DeepSurv | 0.611* (tuned) | 0.611* | +0.000 | Kvamme 2019 (66) |
| SUPPORT | RSF | 0.629 | 0.628* | +0.001 | Kvamme 2019 (66) |
| SUPPORT | DeepHit | 0.600 | 0.639* | -0.039 | Kvamme 2021 (15) |
| SUPPORT | DeepHit | 0.639* (tuned) | 0.639* | +0.000 | Kvamme 2021 (15) |

See Vignette 3 for full analysis. Gap = `mlsurv` performance – target, where a positive number indicates mlsurv outperformed the target. `mlsurv` models are computed using default hyperparameter settings unless marked with an asterisk (*), indicating model hyperparameters were tuned. `mlsurv` tuned with n_trials=100 via Optuna. Kvamme manuscripts used random search across 300 configurations for DeepSurv and DeepHit, grid search across 20 configurations for RSF, and L2 (ridge penalty) tuning across 9 values for CoxPH models. Mean absolute deviation (MAD, mean(|gap|)) was MAD=0.0076 using all `mlsurv` default settings and MAD=0.0031 when `mlsurv` tuned for deep learning models with SUPPORT.

### Principle 2: Multi-Scale Evaluation and Interpretation

A key challenge when using ML survival models is understanding how the model is making predictions. Understanding which groups of patients benefit, which variables matter, and what drives an individual prediction are critical to developing new scientific hypotheses and using ML models well in clinical contexts. `mlsurv` evaluates models along two complementary axes: (1) the observation axis, from population to subpopulation to individual patients; and (2) the feature axis, with individual feature importances as well as feature interaction quantification. Vignette 2 presents a feature importance analysis along with patient-level explanations, and the Chowell immunotherapy case study in Vignette 7 discusses feature interaction and subpopulation analyses.

### Principle 3: Automated Trust and Transparency

Methodological validation, transparent reporting, and reproducibility are essential but rarely built into ML survival software tools. `mlsurv` automates all three: fourteen limitation flags detect methodological concerns (**S2 Table**); a single `generate_report()` command assembles all results, limitations, environment details, and a pre-populated TRIPOD+AI reporting checklist in a standalone, interactive HTML report; lastly, `save_learner()` preserves the analysis state for full-state reproducibility. Vignettes 1, 2, 4, and 7 each produce a generated report. The Chowell case study in Vignette 7 demonstrates limitation flagging for non-proportional hazards and small subpopulation size warnings.

### Case Study: Immunotherapy Response Prediction

Immune checkpoint blockade (ICB) has emerged as a standard-of-care therapy across multiple cancer types (67–72), but as many as half of patients show little to no clinical benefit (73–80). Robust outcome prediction can help identify likely non-responders to avoid the toxicity, cost, and delay of ineffective therapy. We demonstrate the utility of `mlsurv` by predicting cancer patient outcomes when treated with ICB therapy through analysis of the Chowell immunotherapy cohort (23).

#### Cohort and Configuration

The Chowell immunotherapy cohort comprises 1,479 patients across 16 cancer types treated with ICB therapy at Memorial Sloan Kettering Cancer Center from 2015 to 2018 (23) (**Fig 2A**). Recently, a study used this cohort to develop the LORIS (logistic regression-based immunotherapy-response score) machine learning model to accurately predict patient response to ICB therapy using six variables: age, albumin, neutrophil-to-lymphocyte ratio (NLR), tumor mutational burden (TMB), systemic therapy history, and cancer type (58). Results from our survival models are compared to three references: tumor mutational burden (TMB) and PD-L1 expression, the two FDA-approved standard-of-care biomarkers used to predict patient response to ICB, and the published LORIS score for each patient. We train ML models to predict both overall survival (OS, **Fig 2**) and progression-free survival (PFS, **S1 Fig**) outcomes.

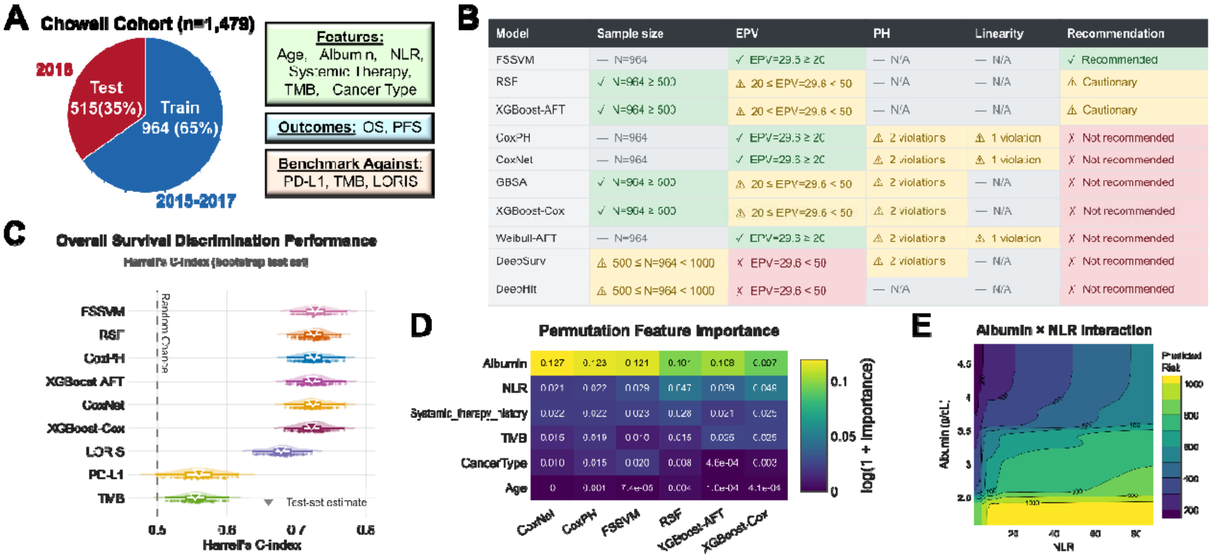

**Fig 2. Chowell immunotherapy survival analysis.** A) The Chowell immunotherapy cohort consists of 1,479 patients treated with ICB therapy. The dataset is temporally split into n=964 (65%) training observations from 2015 to 2017 and n=515 (35%) test observations from 2018 to match LORIS methods. Models are trained on age, albumin, neutrophil to lymphocyte ratio (NLR), systemic therapy history, tumor mutational burden (TMB), and cancer type. Two outcomes are assessed: overall survival (OS) and progression-free survival (PFS). Model performance is benchmarked against PD-L1 tumor proportion score, TMB, and the LORIS score. B) `setup()` runs a dataset audit for sample size, events per variable (EPV), and the Cox assumptions of proportional hazards (PH) and log-hazard linearity. The audit feeds into a model recommendations table which scores each `mlsurv` model as recommended, cautionary, or not recommended. Due to PH and linearity violations within the overall survival (OS) dataset as well as low EPV, only FSSVM is fully recommended, while RSF and XGBoost-AFT are noted as cautionary. C) `evaluate(bootstrap=True)` evaluates the discriminative performance of each OS model using Harrell's concordance index. Bootstrapping the test set produces a performance distribution for each model and inverted triangles denote the performance of the full test set. All OS models show better performance versus benchmarks LORIS, PD-L1, and TMB. D) Permutation feature importance shows albumin consistently ranks as the most important feature for OS prediction followed by NLR. E) The partial dependence plot between albumin and NLR for the RSF model shows how predicted risk varies with the joint effects of both features. The highest risk exists where albumin drops below 2.5 g/dL and NLR is above 10.

The first step in our `mlsurv` pipeline is the `setup()` command. We use the LORIS temporal split—964 patients treated 2015-2017 for training, 515 treated in 2018 for testing—rather than `mlsurv`'s internal splitter to ensure consistency with the LORIS benchmark. `mlsurv` default preprocessing (SimpleImputer, StandardScaler, no feature selection) was used with 5-fold cross validation. Cancer type is registered at set up time as a subpopulation indicator, jointly stratifying the CV folds alongside event status and enabling downstream per-cancer-type evaluation.

Beyond configuring the preprocessing pipeline and CV strategy, `mlsurv`'s `setup()` command computes dataset summary statistics and runs methodological diagnostics to provide model recommendations (**Fig 2B, Fig S1**). Despite sharing the same features, splits, and pipeline configuration, the OS and PFS analyses flagged distinct methodological concerns driven by their different event counts (29.6 vs. 38.7 EPV, respectively) and time-to-event distributions. The OS analysis flagged proportional hazard (PH) violations on albumin and systemic therapy history plus a log-hazard linearity violation on NLR, while the PFS analysis flagged albumin and age for PH violations and showed no linearity concerns. Model recommendations shifted accordingly: on OS, CoxPH and CoxNet were not recommended due to combined PH and linearity violations, while on PFS the lack of linearity violations cleared these models to a cautionary status instead.

### Model Development

Six models (CoxPH, CoxNet, RSF, XGBoost-Cox, XGBoost-AFT, and FSSVM) were fit with a single `learner.run()` command per outcome (OS, PFS). This command executes the full per-model `tune()`, `train()`, `evaluate()`, and `bootstrap()` workflow using the shared `setup()` configuration. Optimal hyperparameters are selected by the highest average C-index score achieved in 5-fold cross validation, and then final models are trained on the full training set. Each fitted model is then evaluated on the held-out test set with bootstrap confidence intervals across both discrimination and calibration metrics.

For models predicting OS, all six `mlsurv` models significantly exceed LORIS performance by an increase in Harrell's C-index of $\Delta C$=0.039–0.050 (bootstrap p≈0.02* for every model). Fast Survival Support Vector Machine (FSSVM (29)), the top-performing model, achieves C=0.728 versus LORIS's C=0.678, while TMB and PD-L1 alone reach only C=0.554 and C=0.560 respectively (**Fig 2C**). Notably, FSSVM was the top recommended model at `setup()` due to Cox violations within the dataset. Its performance here demonstrates the value of matching dataset diagnostics to model assumptions and sample-size requirements.

All OS prediction models were more accurate than the corresponding PFS prediction models. The best PFS model CoxNet achieved C=0.674, with $\Delta C$=-0.054 from the best OS model. A similar gap in performance has been reported in a related study, where OS prediction was more accurate than predicting binary clinical benefit defined by tumor response (81). These results highlight the distinction between prognostication of a patient's overall survival versus prediction of progression during a treatment. In this case study and related work, prediction of progression during ICB therapy is a more challenging target than predicting OS.

PFS-trained `mlsurv` models show parity with LORIS. Four of six `mlsurv` models outperform LORIS though the differences are not statistically significant (best model CoxNet C=0.674 vs. LORIS C=0.664, $\Delta c$=0.009; bootstrap p=0.34). All `mlsurv` models and LORIS perform significantly better than TMB (C=0.576) and PD-L1 (C=0.564).

Subpopulation evaluation across all 16 cancer types reveals substantial model accuracy differences, in part due to small sample sizes. On OS, performance ranges from C≈0.90-0.98 (Breast, n=13) down to C≈0.55-0.62 (Hepatobiliary, n=16), while NSCLC, the largest subtype (n=207), achieves C≈0.69-0.71. Bootstrap confidence intervals reflect the uncertainty around performance estimates across each subtype and metric.

### Feature Analysis and Interpretation

For the models predicting OS, albumin is the most important feature with a permutation importance of around 0.10-0.13 across all models, roughly 5x the next feature (**Fig 2D**). In models predicting PFS, feature analysis showed both TMB and albumin co-led with permutation importance of 0.04-0.06 each. This suggests that low albumin, a marker of cachexia and systemic inflammation, is more prognostic of overall survival, while TMB is more specifically associated with ICB response.

Feature interactions are also relatively consistent across models. Albumin × NLR is the top pair on OS in every model, showing that low albumin paired with high NLR predicts worse outcomes than their marginal effects would indicate (**Fig 2E**). High NLR may reflect tumor-driven neutrophilia, an immune dysregulation that impairs antitumor response.

### External Validation

The `validate()` call evaluates every fitted model across one or more external cohorts. For our analysis, we validate against seven independent ICB cohorts and an OS-only negative control of non-ICB patients assembled in the LORIS study (58). Most cohorts show a modest discrimination drop from the internal test set $\Delta c \approx 0.04$–0.10 across all models, consistent with expected cross-cohort variation.

On the negative control cohort of non-ICB patients (OS only), `mlsurv` models largely retain their accuracy. `mlsurv` models achieve a C-index range of 0.67-0.70, only 0.02-0.06 below ICB cohort performance, indicating that much of the discriminative ability reflects general prognostic signal rather than ICB-specific effects.

### Reporting

The `generate_report()` command bundles all results into a standalone HTML. It includes a pre-populated TRIPOD+AI checklist (19) that provides full or partial information on 29 of 52 items, simplifying methodological reporting. Next, `patient_report()` produces a patient-specific report HTML. As an example, patient 208 from the hold-out test set is placed at the 91st risk percentile of the training cohort (high risk) and shows a median predicted survival of 1.2 months [bootstrap 95% CI 1.0-1.9 months]. A SHAP waterfall plot shows individual feature contributions to the patient's risk score and k-nearest neighbor tables by risk score and feature-space distance show similar patients within the training set.

### Case Study Scientific Findings

Our case study is the first to compare OS and PFS survival ML models with a classification ML model for ICB outcome prediction in the large, public Chowell dataset. Using `mlsurv`, we find that OS prediction is more accurate than PFS prediction, in accordance with prior studies (81,82). OS predictions may be more accurate than PFS predictions for several reasons. Progression is often challenging to measure well, leading to inexact measures that make prediction difficult. In addition, the Chowell dataset's patient features may be better suited to prognostic (OS) prediction than therapy response prediction even though they were first used to predict response. Next, we showed that LORIS matches PFS model performance but is less accurate for OS prediction versus time-to-event OS models. These results suggest that ML classification models that predict therapy response cannot be used for predicting other aspects of patient outcomes. We also observed feature interaction effects between albumin and NLR in our OS prediction models, where the adverse prognostic effect of low albumin was amplified in patients with high NLR. Patients exhibiting both risk factors had substantially poorer OS than would be anticipated based on the independent effects of each biomarker, indicating a clinically meaningful interaction. Follow up work to externally validate this interaction and, if validated, understand its biological mechanisms would be valuable. Each of these findings were easily uncovered by simple `mlsurv` commands and automated comprehensive reporting.

## Future Development

Planned near-term development includes integration with the Galaxy computational workbench to broaden access to researchers with limited informatics skills and support reproducible workflow sharing (83–85). We also plan to expand methodological scope to support left/interval censoring and offer a wider set of recalibration methods. Long-term development will focus on addressing the limitations discussed in the previous paragraph. Support for multi-modal input expansion for imaging and free text will be developed using a combination of external embedding pipelines and native architectures. Federated and multi-site learning will be implemented to enable site-local training with aggregated coefficient updates, preserving data privacy while extending the effective training cohort across institutions.

### Limitations

`mlsurv` has three important limitations. First, `mlsurv` is intended primarily as a research tool. Translating a model developed in `mlsurv` into clinical use involves downstream steps that fall outside the scope of the software and are best addressed within institutional and regulatory processes. These steps include regulatory-grade model validation, model integration into clinical workflows with appropriate display of model predictions to guide clinical decisions, and post-deployment performance monitoring. Second, input data to `mlsurv` is currently limited to tabular format. Raw imaging and free-text data require external embedding pipelines to be converted into tabular features before entering the `mlsurv` workflow. Third, `mlsurv` assumes a single training cohort; federated and multi-site learning protocols are not supported within the current framework.

## Conclusions

Survival analysis is a foundational tool of biomedical research, and machine learning methods for survival analysis are increasingly used to capture high-dimensional and nonlinear patterns that classical Cox regression cannot. Conducting these analyses requires methodological rigor, multi-scale evaluation, and transparent reporting. Several Python software packages provide survival model implementations, but these are limited to model training and prediction, leaving the many surrounding workflow steps undefined. `mlsurv` closes this gap by integrating dataset diagnostics, model development and evaluation, methodological flagging, comprehensive interpretation, and automated reporting into a single workflow. We validated `mlsurv` against published results for numerous public datasets and demonstrated a mean absolute discrepancy below 0.01 in concordance index metrics. With our case study, we then demonstrated a full `mlsurv` pipeline through dataset auditing, comprehensive evaluation across models and subpopulations, feature importance and interaction analysis, external validation, and both cohort- and patient-level automated reporting.

`mlsurv` fills three critical gaps in the ecosystem of software tools for survival analysis: (1) ensuring methodological rigor through strong guardrails; (2) enabling systematic model benchmarking; and (3) mitigating risks associated with AI-generated code. A main gap filled by `mlsurv` is enabling researchers to perform rigorous end-to-end survival ML analysis. At every step, `mlsurv` automates the use of best practices that would otherwise demand expertise across disparate tools and methods. This includes use of automated data validity checks, model assumption tests, and optimization of all survival models. A second gap that `mlsurv` fills is performance comparison across many survival models, including multiple types of ML models. This benchmarking is an important but often ignored step in performance optimization, as no single model has emerged as the consistent best choice for survival analysis (20–22). `mlsurv` also supports custom models (see vignette 4), enabling model developers to compare the performance of new models to the existing suite of `mlsurv` models. The third gap that `mlsurv` addresses is potential methodological and coding errors that arise from the use of “vibe coding”

with large language models and artificial intelligence (AI) agents (9,10,86,87). Researchers can vibe code analysis scripts in seconds, but these scripts can include methodological errors and skip important tests. `mlsurv` uses built-in best practices and automated reporting to mitigate these exact risks. For these reasons, we believe there is substantial value of best practice data analysis software packages like `mlsurv` in an era of AI-generated code.

## List of Abbreviations

- AFT: accelerated failure time
- AI: Artificial Intelligence
- AUC: area under the curve (in this case, the receiver operating characteristic curve)
- C-index: concordance index
- CLI: command line interface
- CoxPH: Cox proportional hazards
- CSV: comma-separated values
- CV: cross-validation
- EPV: events per variable
- FLCHAIN: Free Light Chain dataset
- FSSVM: Fast Survival Support Vector Machine
- GBSA: Gradient Boosted Survival Analysis
- GBSG2: German Breast Cancer Study Group 2 dataset
- GND: Greenwood-Nam-D’Agostino test
- HTML: hypertext markup language
- IBS: integrated Brier score
- ICB: immune checkpoint blockade
- IDI: Integrated Discrimination Improvement
- LORIS: logistic regression-based immunotherapy-response score
- MAD: mean absolute deviation
- METABRIC: Molecular Taxonomy of Breast Cancer International Consortium dataset
- ML: machine learning
- NLR: neutrophil-to-lymphocyte ratio
- NRI: Net Reclassification Improvement
- OS: overall survival
- PD-L1: programmed death-ligand 1
- PFS: progression-free survival
- PH: proportional hazards
- RMST: restricted mean survival time
- RSF: Random Survival Forest
- SHAP: Shapley Additive exPlanations
- SUPPORT: Study to Understand Prognoses Preferences Outcomes and Risks of Treatment dataset
- TCGA BRCA: The Cancer Genome Atlas Breast Invasive Carcinoma dataset
- TMB: tumor mutational burden
- TPE: Tree-structured Parzen Estimator
- TRIPOD+AI: Transparent Reporting of a multivariable prediction model for Individual Prognosis Or Diagnosis, plus Artificial Intelligence

## Declarations

### Ethics approval and Consent to Participate

Not applicable.

### Consent for Publication

Not applicable.

### Availability of Data and Materials

All data underlying the findings in this study are publicly available and require no special access. This study used only previously published datasets; no new data were generated. All `mlsurv` source code is available at https://github.com/goeckslab/mlsurv. We provide cached data files for reproducibility of our analyses within our GitHub project repository at https://github.com/goeckslab/mlsurv/tree/main/examples/data. Dataset citations are documented in **Table 4** and within each vignette where they are used.

- Project name: `mlsurv`
- Project home page: https://github.com/goeckslab/mlsurv
- Operating system(s): Platform independent
- Programming language: Python, CLI
- Other requirements: Deep models are GPU-optional
- License: MIT
- Any restrictions to use by non-academics: None

### Competing Interests

The authors declare that they have no competing interests.

### Funding

This work was supported by the National Institutes of Health awards U24CA284167 and U24HG006620 (JG, www.nih.gov) as well as Moffitt Cancer Center (JG, www.moffitt.org).

### Author Contributions

AFP and JG conceived and designed the study. AFP, JQ, PCML, and KD created the software. AFP, INB, and TJ analyzed the data. AFP drafted the manuscript with edits from PCML and JG. All authors read and approved the final manuscript.
